# “My work? Well, I live it and breathe it”: The seamless connect between the professional and personal/community self in the Aboriginal and Torres Strait Islander health sector

**DOI:** 10.1186/s12913-020-05804-3

**Published:** 2020-10-23

**Authors:** Michelle Dickson

**Affiliations:** Sydney School of Public Health, Faculty of Medicine and Health, The University of Sydney, Edward Ford Building, Sydney, New South Wales 2006 Australia

**Keywords:** Aboriginal and Torres Strait Islander, Indigenous, Professional boundary, Indigenous research methodology, Professional practice, Personal/professional

## Abstract

**Background:**

Australian Aboriginal and Torres Strait Islander health professionals often juggle the challenges of working and living in the same community in ways that are positive for both themselves and their clients. This study specifically examines the strategies Aboriginal and Torres Strait Islander health professionals have developed to enable them to feel empowered by the sense of being always visible or perceived as being always available. Findings provide examples of how participants (Team Members) established a seamless working self, including how they often held different perspectives to many work colleagues, how Team Members were always visible to community and how Team Members were comfortable to be seen as working when not at work.

**Methods:**

This qualitative study engages an Indigenous research methodology and uses an Indigenous method, PhotoYarning, to explore lived experiences of a group (*n* = 15) of Aboriginal and Torres Strait Islander health workers as they worked in the Australian health sector.

**Results:**

The analysis presented here comes from data generated through PhotoYarning sessions. Team Members in this study all work in health care settings in the communities in which they also live, they manage an extremely complex network of interactions and relationships in their daily working lives. They occupy an ambivalent, and sometimes ambiguous, position as representing both their health profession and their community. This article explores examples of what working with seamlessness involved, with findings citing four main themes: (1) Being fellow members of their cultural community, (2) the feeling of always being visible to community as a health worker, (3) the feeling of always being available as a health worker to community even when not at work and (4) the need to set an example.

**Conclusions:**

While creating the seamlessness of working and living in the same community was not easy, Team Members considered it an important feature of the work they did and vital if they were to be able to provide quality health service to their community. However, they reported that the seamless working self was at odds with the way many of their non-Indigenous Australian colleagues worked and it was not well understood.

## Background

### Locating myself in this research

As an Aboriginal and Torres Strait Islander^1^ (Koori^2^) person I will follow cultural protocol and introduce and position myself in this article:

*I am Michelle Dickson. My family are from Ngarigo lands (in the Snowy Mountains region in New South Wales, Australia) and Darkinjung lands (on the Central Coast of New South Wales, Australia). Sadly, like many of my Aboriginal and Torres Strait Islander friends and colleagues, both sides of my family suffered the impact of what is now referred to as The Stolen Generation* [1]*. Removing Aboriginal and Torres Strait Islander children from their families and communities was made official under various government laws and policies in Australia until 1969. I was born in 1967, so this was still happening in my own lifetime. I was born on Cammeraygal lands (north of the harbour in Sydney, New South Wales, Australia) and for much of my life I have lived and worked on the lands of the Eora nation in Sydney. I have four (grown up) children of my own and I have many nieces and nephews. I am a senior lecturer in the University of Sydney’s Sydney School of Public Health.*

Important to this research is how working in ‘culturally safe’ ways is understood and applied in the Australian Aboriginal and Torres Strait Islander health sector [2, 3]. Cultural safety, informed by the seminal work of Ramsden [4, 5] and Papps and Ramsden [6], can be understood as a way of working at individual and institutional level to “create a safe space for an encounter with patients that is sensitive and responsive to their social, political, linguistic, economic, and spiritual realities” [7] pp157–158). However, relevant to my research is the understanding that cultural safety moves beyond focusing on the skills and competence of an individual health professional to include “analyzing power imbalances, institutional discrimination, colonisation and colonial relationships as they apply to health care” [8] p3).

The Aboriginal and Torres Strait Islander health professional often works in partnership with other (non-Indigenous) health professionals as they provide health services to Aboriginal and Torres Strait Islander clients and communities. This brings together ways of delivering health service that comprises Western and Aboriginal and Torres Strait Islander ways of working. In many cases health services remain dominantly structured and delivered according to a Western biomedical model of care that is not necessarily “sensitive and responsive to [Aboriginal and Torres Strait Islander] social, political, linguistic, economic, and spiritual realities” [7] p157–158). Effective interprofessional collaborations between Aboriginal and Torres Strait Islander and non-Indigenous Australian health professionals are one mechanism for building cultural safety in health service delivery [9, 10]. Developing a collaborative way of working often requires sustained effort and time [11] but can produce a working relationship that is built on trust and has a deeper understanding of Aboriginal and Torres Strait Islander ways of working that can increase cultural safety for health practitioners and clients [12].

Aboriginal and Torres Strait Islander health professionals have long been positively regarded for their role as cultural brokers, guiding their non-Indigenous colleagues and ensuring best practice of care for their Aboriginal and Torres Strait Islander clients [12–14]. Developing an understanding of Aboriginal and Torres Strait Islander health and health practice is considered to be culturally safe [15] and ultimately provides scope for non-Indigenous Australian health professionals to better meet the needs of their Aboriginal and Torres Strait Islander clients: “…a culturally safe practitioner uses his or her knowledge to navigate the system and apply flexible processes to ensure that they meet the cultural needs of Aboriginal and Torres Strait Islander patients” [12] p176). Of particular relevance to this research is Sherwood’s work [3] that further explores cultural safety within an Aboriginal and Torres Strait Islander Australian health care context. Her work argues that the path to working with cultural safety involves health professionals using critical reflexivity to explore their worldviews and assumptions and undertaking deep thinking about how those have potential to impact on power imbalances and safe, equitable practice in health service delivery.

Mercer [10]  states that the responsibility for building collaborative working relationships between Aboriginal and Torres Strait Islander and non-Indigenous Australian health professionals needs to be shared between health services and the individuals concerned; however Abbott, Gordon, and Davison [16] place more responsibility on to health services and work environments, reporting that some Aboriginal and Torres Strait Islander health professionals indicated that their work was “strongly affected by the setting in which they work” ([16] p157). Those healthcare settings included a dominant, Western model of healthcare service design and delivery that frequently privileged a Western, biomedical model of care that embodied a power imbalance between Western and Indigenous knowledges and healthcare provision. In addition to acknowledging the important role health professionals should play in building collaborative working relationships in Aboriginal and Torres Strait Islander health service provision, Mercer [10] highlights the commitment required by health services and the health workforce policies that inform practice, suggesting that “the experience of each partner in the arrangement is heavily intertwined and influenced by the culture and support offered from within the workplace and across the workforces” ([10] p327). For the Australian health sector to provide culturally safe [4, 17, 18] services that meet the needs of Aboriginal and Torres Strait Islander peoples of Australia it needs to better engage with Aboriginal and Torres Strait Islander ways of being, knowing, doing and seeing. This includes respecting and valuing Aboriginal and Torres Strait Islander knowledges shared by land and waterways, flora and fauna, the environment, and spiritual and cultural systems; gained through “listening, sensing, viewing, reviewing, reading, watching, waiting, observing, exchanging, sharing, conceptualising, assessing, modelling, engaging, applying” ([19] p207). Engagement with Aboriginal and Torres Strait Islander knowledges subsequently informs and influences ways of being (for example, our relationships and interactions with people) and ways of doing (for example, how we work or how we deliver health services), thus contributing to culturally safe ways of working [20]. Sherwood states that working with “cultural safety means Aboriginal people feel respected, and power dynamics are acknowledged and addressed” ([3] p172); culturally safe health services demonstrate engagement with (and respect for) Aboriginal and Torres Strait Islander ways of being, knowing, doing and seeing and embed that in policy and practice.

### Community-based health workers improving client engagement with, and experience of, health service provision

It is now acknowledged that health workers who either live and work in the same community as, or share a cultural connection with, their health clients can enhance health systems, in part because of their community engagement, community knowledge and cultural connections [21, 22]. In an Australian context we have evidence that some health services (and their clients) have benefitted from employing Aboriginal and Torres Strait Islander health staff who not only share cultural backgrounds but also share the community with their clients by living and working (community-based) in the same place. Aboriginal and Torres Strait Islander women experienced improved health outcomes and increased engagement with maternity services that employed Aboriginal and Torres Strait Islander health staff and health students, partly due to familiar communication styles, community familiarity and relationships that extended beyond “the boundaries of a clearly defined professional relationship” ([23] pp3–4). Several studies from the alcohol and drug sector also highlight the importance of familiarity [24–27].

### Unofficial roles and additional responsibilities

In addition to their clinical and health-based roles, Aboriginal and Torres Strait Islander health workers often maintain close connections with community cultural, social and political knowledge [10, 13], and perform additional roles, responding to unique client and community needs by liaising between community and health services, performing community visits and engagements, and supporting clients from the community who might not directly be within their own work caseloads [13, 27, 28]. While some health services have positions, such as Aboriginal liaison officers, that recognise these duties as part of the job, other services consider these activities as informal and often do not recognise them as part of the position descriptions of an Aboriginal and Torres Strait Islander staff member [25]. Even though many of these ‘unofficial’ roles might not be documented in position descriptions, they are often seen by the Aboriginal and Torres Strait Islander health staff themselves as being essential to the work they do, albeit at times challenging: “Bearing the load of community expectation can be very tiring when combined with the responsibilities of work and family. We cannot go out after work and relax, as community members may want to unload their problems on us” ([28] p530).

### Health services and health workers’ community engagement

While it is documented that health care can be improved through genuinely improving engagement and partnership with Aboriginal and Torres Strait Islander clients and their communities, there remains a need to adopt a better way of achieving such engagement and partnership [21, 29]. Willis et al. suggest a power shift is needed that

…requires a shift from expecting Aboriginal patients to adapt to the expectations of the health service to the health service being more inclusive, collaborative and flexible in responding to the needs of Aboriginal people in ways that are respectful and more likely to build trust and strengthen relationships” ([30] p10).

Often working relationships between Aboriginal and Torres Strait Islander health professionals and their clients align with “cultural and social structures based on family, kinship and community relationships” ([23] p3) that also determine how Aboriginal and Torres Strait Islander people interact with each other.

A recent study noted that some Aboriginal and Torres Strait Islander health professionals feel “restricted in their capacity to practice in their communities” ([31] p68), partly due to their health service not understanding that “Indigenous practice” and “best practice” could coexist ([31] p68); the health professionals were not restricted by personal factors but by the lack of workplace support to work this way. In another study, Aboriginal and Torres Strait Islander health professionals considered developing respect for Aboriginal and Torres Strait Islander ways of practice in health service provision to be essential to improving the health service experience for Aboriginal and Torres Strait Islander clients; this was also considered as a means of increasing positive health outcomes [32]. However it was also acknowledged that “sufficient organizational commitment” ([32] p11), understanding and respect is required to integrate Aboriginal and Torres Strait Islander ways of practice into the dominant Western health care system.

### Roles and identities

For this research, my working definition of identity was that it is “contextually specific, fluid, a conjoint construction created … in interaction with others” ([33] p34). As a Koori researcher I choose to privilege two theories as an overarching theoretical framework for this article. Cultural Interface Theory [34–37] helps explain how Team Members^3^ work fluidly in a space between a dominant Western health system and an Aboriginal community with different expectations and needs, and Indigenous Standpoint Theory [36, 38–41] that helps us understand how Team Members enact their Aboriginal and Torres Strait Islander ways of being, knowing and doing in the work they do with clients as they avoid separation between the work they are doing in community as a health professional and their membership of the same community.

### Balancing roles and identities

Studies have identified both benefits and challenges in maintaining multiple workplace connections and relationships [42, 43], reporting on the benefits and challenges of maintaining multiple relationships and professional connections when working in a rural context where a health service provider is often also a member of other social and cultural parts of the same community. It cannot be assumed that clean boundaries can be maintained based on the assumption that a health professional and a client live in separate worlds. Boundary work becomes increasingly difficult to maintain when the worlds of the client and health professional are the same, or shared. For example, this difficulty has been explored in the context of mental health clinicians working in rural Australia [44], in rural and remote community work [45], in rural social work [42, 43], and in remote Australia [46], when clients and clinicians share the same cultural background [23].

Stets and Burke [47] remind us that people often simultaneously perform multiple, possibly conflicting or complementary, roles. Aboriginal and Torres Strait Islander health workers who live and work in the same community face complexities. Their identities may be based on their role as health professionals within a community context in which they also have a role (or roles), while their workplace also provides a social group or context they belong to. They will prioritise one role or another based on its importance to their identity, and this may change. Thus, for example, Team Members who chose not to drink in public may be seen as prioritising their health worker role (modelling good health behaviours around a community health issue of concern) and also ranking their commitment to community above their role as an employee or colleague (drinking with colleagues to bond, unwind or celebrate a shared win). However, as the findings show, for most Team Members their sense of themselves as health worker and as a community member were equally important and blended seamlessly together.

## Methods

### Theoretical framework

This study uses a theoretical framework Indigenous^4^ methodology that privileges Aboriginal and Torres Strait Islander ways of being, knowing and doing [19] and was undertaken as part of a doctoral study [48, 49]. Linda Tuhiwai Smith’s decolonising theories [50] and Moreton Robinson’s Indigenous Women’s Standpoint Theory provided a foundation upon which I built my own Indigenous Standpoint in this research [40], allowing me to undertake this study by centering my experience of Aboriginal ways of knowing, being and doing. Nakata’s Cultural Interface theory [35] provided a theoretical framework through which to explore how Aboriginal and Torres Strait Islander peoples worked with the dominant Western health system in Australia. The Cultural Interface explores the contested knowledge space between Indigenous and Western knowledges. As a Koori researcher working at a Western University, I work at the Cultural Interface and so too did the Team Members in this study, as later shown in my findings. Team Members gave me accounts of their experiences of working at the Cultural Interface; working as Aboriginal and Torres Strait Islander health professionals who uphold Aboriginal and Torres Strait Islander knowledges and ways of working and do so within their professional roles in health systems and services that operate (mostly) from a western base of knowledge and operation. While some literature refers to the “differences” between these knowledge systems as ontologically and epistemologically “irreconcilable” (Russell, 2005, ^p.166^); Team Members’ data presents both examples of the challenges faced when contesting the knowledge space when working at the Cultural Interface, and, importantly, examples of opportunities for the health system and for their colleagues to embrace. However, differences at the epistemological and ontological levels can make it difficult to establish an effective working space at the Cultural Interface, particularly in workplaces that dominantly align with a Western knowledge system.

### Ethics

I commenced my PhD at Macquarie University in Sydney, New South Wales, Australia and later completed at The University of Sydney, Sydney, Australia. This study was part of the larger doctoral work and was granted ethical approval through the relevant University’s Human Research Ethics Committee (HREC) and followed both Indigenous ethical and cultural protocols and Western research guidelines [51–53].

### Recruitment and team members

An expression of interest, using participant information sheets and an expression of interest notice approved by the HREC was distributed throughout my existing Aboriginal and Torres Strait Islander networks in government, non-government and community-controlled health services that largely welcomed this research. The recruitment criteria required a person to identify as Aboriginal and Torres Strait Islander, be employed in a health related role, be interested in engaging in research about working in Aboriginal and Torres Strait Islander health and be available, over 6–12 months to engage in the research. Each Team Member met with me between two and four times. Fifteen Aboriginal and Torres Strait Islander people were recruited; all worked in health services that provided health and wellbeing services to Aboriginal and Torres Strait Islander peoples. Ranging from 24 to 52 years of age, nine worked in Aboriginal Community-Controlled Health Services, four in non-government health settings and six worked in public (government) health services. Team Members worked within five Australian states or territories. One worked in a remote community, two in a rural setting, six in regional communities and six in an urban context. The average number of years Team Members had worked in Aboriginal and Torres Strait Islander health was 13, with the longest term of employ being 30 years.

## Methods

I developed a new Indigenous research method, PhotoYarning [49] as part of my doctoral research and that method was used in this study. PhotoYarning has Aboriginal and Torres Strait Islander epistemology and ontology at its core and employs Yarning [54–59] about photographs (PhotoYarning) taken by Team Members on a camera provided by the researcher. It values a person’s own expertise and privileges their life experiences through a process that focuses on photographs they have taken to describe significant themes, events, or phenomena. Described in detail elsewhere [49], PhotoYarning, in summary, has five stages that move the research process through data generation to a process of analysis that includes small group analysis (involving the Team Members) and my own solo analysis. Team Members took photographs of images that represented themselves at work and elements of their experiences at work. The photographs formed the basis of individual PhotoYarning sessions, held between Team Members and me. With consent, I recorded and transcribed the PhotoYarning sessions. Three small groups, each comprising four Team Members, then came together for group PhotoYarning sessions and small group co-analysis was also undertaken. The small groups decided what photographs best illustrated the identified key themes and those chosen have been used as data in this publication. Following this series of small group analysis sessions, I completed solo analysis. The analysis used in PhotoYarning follows Braun and Clarke’s method of thematic analysis [60]. Team Members (participants) chose pseudonyms to uphold anonymity and those pseudonyms are used throughout this manuscript.

## Results

The following results focus on how Team Members established a comfortable, seamless connect between their professional identities and personal/community identities which I have called the ‘seamless working self’. Team Members described being able to work in a seamless way as meaning they were able to maintain their responsibilities and roles as both Aboriginal and Torres Strait Islander community members and Aboriginal and Torres Strait Islander health professionals. Team Members provided examples of what this seamless connect involved, citing four main aspects: (1) being fellow members of their cultural community, (2) the feeling of always being visible to community as a health worker, (3) the feeling of always being available as a health worker to community even when not at work and (4) the need to set an example.

### Being a fellow member of my cultural community

**Fig. 1:**
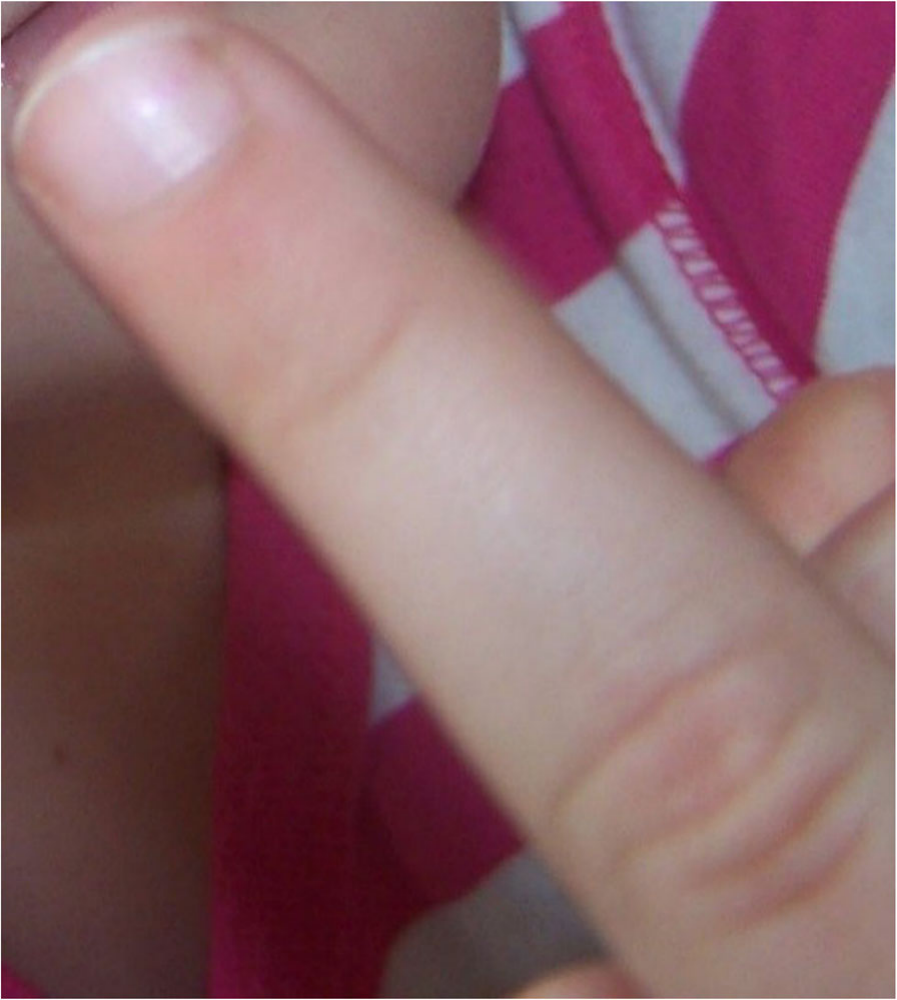
The royal finger

Tia^5^ explained that she had invited her new manager over to her home for dinner and described how she and her new manager had been approached by a client ‘Kev’ while shopping together for dinner items (Fig. 1):

*Sometimes my work mates get the finger from a client and then turn and ask me what they have done. I live in a small community, you know, everyone knows everyone. Our health service had a new manager and she was coming to my place for dinner. She was with me in the shop when “Kev” came over to me and started to talk.*

*I introduced Kev to my new manager and then Kev started to tell me some news about his health. My new manager was shocked and tried to grab my arm and pull me back to my shopping. She said something like “Nice to meet you Kev, well, make an appointment with Tia and you can talk about it then.” She kinda gave him the finger, like this photo.*

Tia continued to talk about the impact that her manager’s action had on Kev, and on her:

*Kev noticed she wanted me to move and got real embarrassed, apologised and then walked on. I was furious. Kev vanished before I could go yarn with him again. I looked at my new manager and said, “What did you do that for?” She just said “Well, you are doing your shopping and after all he is only a client.”*

*Right then I knew she wouldn’t last in this community, in this job. I had to work hard to get over the shame that caused both me and Kev. I had to build up all that trust [with Kev] all over again. The manager, she didn’t learn a thing from all that.*

In this PhotoYarn Tia highlighted how she became angry at her new manager who, Tia believed, separated her personal self and professional self. Her manager, a non-Indigenous person, said to Tia that Kev “is only a client”, and while she too lived and worked in the same community and would be considered a member of the broader community, did not adopt the same seamless approach to her work/personal identities as did Tia. Tia explained that Kev saw her (Tia) as something more than simply his health worker, he also saw her as a fellow member of the cultural community. Tia explained that she knew Kev’s family, and they knew hers and as such they had professional connection that was interwoven with a range of cultural and community obligations, roles, and responsibilities that she had to uphold.

This example illustrates the cultural complexity faced by Tia as she worked and lived in the same community; she had developed a strategy to maintain her professional identity alongside her community identity. Tia could clearly see the impact her manager’s reaction had on her client. Tia named that both she and her client felt Shame, a word broadly used in Aboriginal and Torres Strait Islander communities to refer to a personal, public, family or social display that breached accepted Aboriginal and Torres Strait Islander “norms”. These might include, but are not limited to, shameful behaviour, rudeness, embarrassment, lack of respect or displays of self-importance. Tia expressed that this Shame would create difficulties for her client (who, she believed, felt he had done the wrong thing), for her (she challenged her new manager’s behaviour and realised the episode meant she (Tia) would need to rebuild trust with the client). She also knew it would create difficulties for her manager, whose actions, in Tia’s opinion, might make it hard for her (the manager) to be accepted in community. This shows Tia working in the Cultural Interface [36] as she is familiar with both sets of cultural expectations and needs to make decisions about who or what to challenge, accommodate or repair. Because Tia knows both cultural spaces she can see the space that is contested and can develop strategies for working in that contested space by engaging with her clients in a friendship-like way [61] while maintaining appropriate boundary work.

Marlene described a similar incident with a colleague who was worried about bumping into a client outside of work, suggesting that she and Marlene avoid him:

*I have been walking at lunchtime with someone from the office here and we see a client coming our way. I said "Hey, it's "Charlie" and my workmate says, "Maybe we should cross the street". I couldn't believe it. And Charlie could clearly see us. She was really panicked about seeing that fella. I laughed because I thought she was joking, but then saw her face. I couldn't say a word to her. I felt sick that someone who worked with this guy (Charlie) could even think about not seeing him, ignoring him.*

**Fig. 2:**
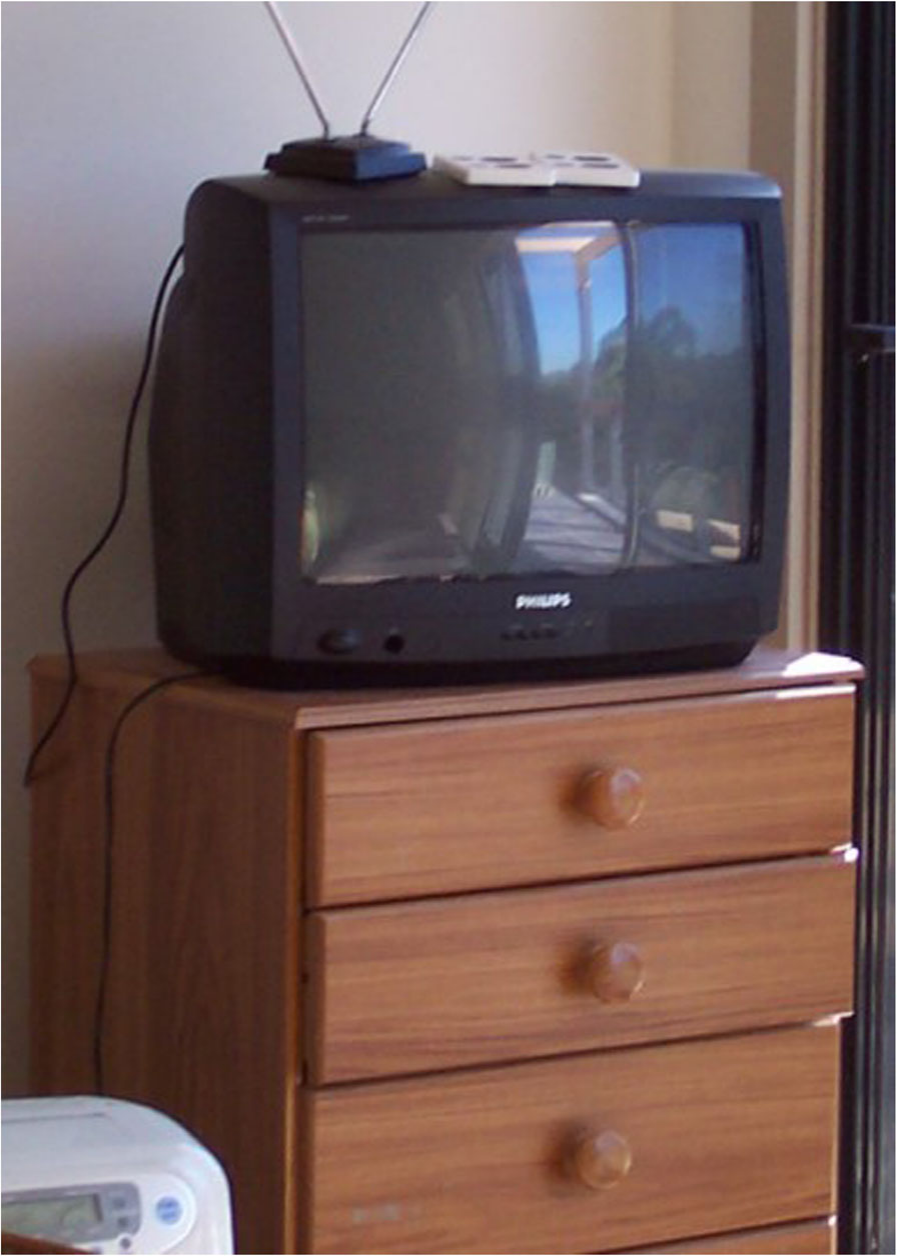
Always seen

Rather than avoidance, Marlene’s priority was to engage with Charlie. In this quote Marlene refers to Fig. 2 (*Always seen)*, that she used here in her PhotoYarning:

*…We kept walking, and she [colleague] really pulled back a bit, but I kept my pace. Charlie saw us - shit, he could have run or crossed the road so he didn't talk to us, but y'know what?...His face lit up with a big smile. He started to walk a bit faster, y'know with a bit of a step in his walk, and said "G'day ladies. How's things?" I smiled back and said "Yeah, good Charlie, just out getting' some lunch. How about you? How's your day?" Charlie than said “Ah, well, y'know a bit of this and a bit of that. All good though." Charlie looked at [colleague], and she said nothing.*

*I didn't even look at her I was so shocked by her first reaction. My concern was Charlie- I didn’t want him to think I was like her, or pick up some bad vibes, y'know?*

*We might have been on our way to lunch but Charlie still saw us in that TV reflection- my colleague [non-Indigenous] might have had the TV switched off and thought she had a blank screen but me, well I know there is always a reflection on my TV screen.*

Unlike her colleague, Marlene described always being “on the screen” and visible in community and to clients, demonstrating her awareness of community practices and expectations, and her sense of being a community member who is obligated to other community members in need. However, Marlene is also aware of the professional practices of her colleague that support turning off or being somewhat invisible during breaks at work or after work. Marlene lives and works in the contested space of the Cultural Interface and understands both ways of being, knowing and doing; she has developed strategies that allow her to maintain her professional responsibilities and her community responsibilities concurrently; she used friendship-like communication with her clients [49, 61] to communicate with empathy while maintaining boundaries that were respected by both her clients and herself. Being on an official lunch break did not make Marlene feel invisible to clients; she had seamless visibility and has friendship-like ways of working to support her seamlessness.

### The feeling of always being visible to community as a health worker

Marlene PhotoYarned about the photo she had taken, *Always seen* (Fig. 2), and described the reflection on the TV screen as being like her identity at work and in community. The photograph allowed her to Yarn about how she always felt visible to community, and that visibility extended to all parts of her life in the community in which she lived and worked:

*See this TV screen? Well it is off but you can still see an image on the screen. That’ how I am – I might finish up my day at work but people can still see me in community, see who I am and what I do. And they see me as many things- their family member, the health worker, the person doing her shopping one night and talking to them at the clinic the next morning… [extended pause]… Like this reflection on the screen, people can always see me. I can't turn it off. Sometimes I can put it in the background a bit, but it is still there, ready to pop back into my head anytime. Don't get me wrong. I love my job, love what I do. But sometimes I'd like to go to sleep and not be worrying is my client is going to binge overnight, or if another client is going to stay dry over Christmas. Things like that.*

Marlene says that she is always being seen by others, whether she is at work or away from work, and indicates the difficulty of being in that position, stating “I can’t turn it off”. Being always visible, and working seamlessly, does create an emotional burden for Marlene who finds it hard to stop worrying about her clients overnight. Not being able to turn off from work has been recognised as a contributing factor to burn out [62] and while Marlene identifies this as a challenge for her she also had established strategies that prevented her from being overwhelmed completely by the emotional burden of her work.

In the extract below we can see Marlene has clearly identified that she had a different philosophy about being visible to community to her colleagues. She indicates that her colleague leaves his work at work and then doesn’t notice clients in the community outside, and clients don’t notice him outside of work. She suggests this process made the clients invisible to him, but insisted that the situation was different for her, as she was always visible and always saw the clients as visible too:

*Actually although I say it worries me I wouldn't want to be like [colleague] who pushes his chair under his desk, and leaves it all there as he walks out that office door. Y'know, at least I know the clients by name if I see them in the street, or at the school or the shops. [Colleague’s name] pretends he doesn’t see them- like clients are invisible as soon as they leave here, like a ghost. But like things on this TV screen, people can been always be seen. And I know I am always on that screen.*

Marlene’s awareness of working in the Cultural Interface is evident here; she is aware of both dominant work protocols and practices (leaving work at work at the end of the day, not ‘seeing’ clients outside of work) and of community protocols and practices (actively engaging with other community members regardless of the context, being available for community). Her awareness affords her the privilege of making choices and developing strategies so she can uphold both spaces.

**Fig. 3:**
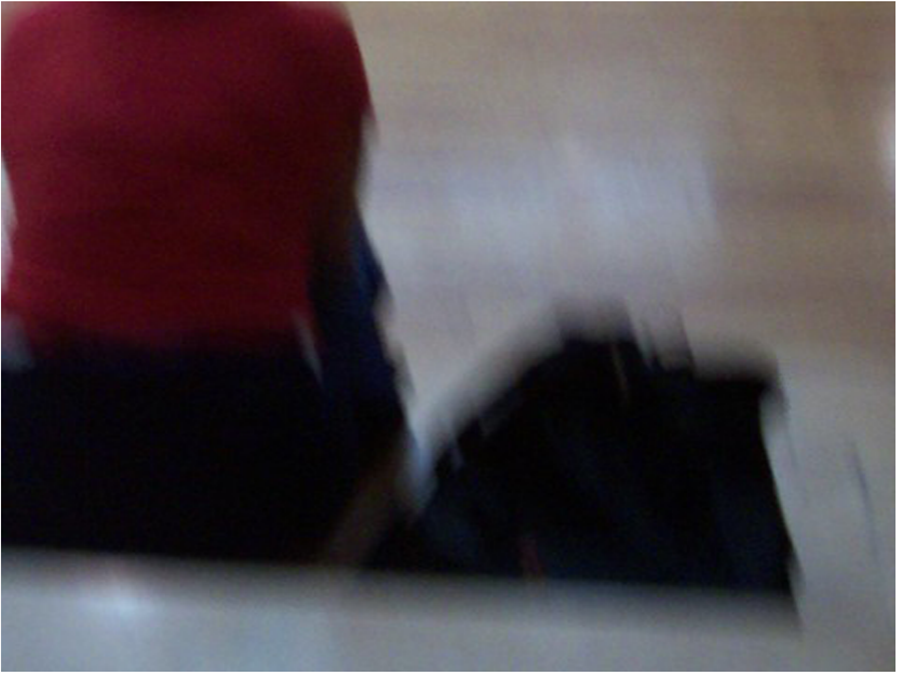
I don’t get blurred like this. I can be seen

Helen took Fig. 3 (*I don’t get blurred like this*) and used it to PhotoYarn about the open connection between her professional self and personal identity in the community in which she worked and lived. She described working with seamlessness; finding a place at which she was completely comfortable being identified as the health worker, regardless of the time or location:

*This is a client of mine – but I took a real blurry picture of her. She said it was fine to photo her, as long as I don’t see her face so we thought it was a good idea to take one from the back, and make it real blurred…I work closely with this client and as we talked about how to take this photo she said that everyone in community knows her anyway so she didn’t mind a front on photo, but people outside the community don’t, so she wanted to stay blurred. That made me smile because she and I were so alike – everyone in community knows me too, they see me and know I am the health worker who also lives here. There’s no blurring who I am.*

Helen was comfortable knowing that she was identified as a health worker even when she was not ‘clocked on’ during working hours, community knew her to be that person and knew as a community member that her professional role would always be seen as part of her ‘complete’ self, that her personal and professional selves are connected:

*Even when I am not clocked on at work I am still clocked on- people don’t look at me differently, they just see the part of me that works as a health worker and it’s like that never goes away or gets blurry. I reckon even a photo of me taken from behind would be recognised in community!*

According to Helen, she might not be constantly seen as the health worker if she had another job elsewhere. She believed that part of feeling comfortable being seen in her professional identity all the time was due to the fact there was so much contact with clients, even in a community setting. There was no room for anonymity or for a differentiation between her professional and personal/community identity because they were one and the same. Helen’s lived experience of the Cultural Interface provided her with scope and awareness to adopt ways of being, knowing and working that were seamless, that aligned the contested space between dominant professional ways of working and Aboriginal and Torres Strait Islander ways of being, knowing and doing:

*But if I were to work in another place well, they wouldn’t know me as I walked out of the office and down the street would they? I might never see a client again if a worked in another place, but here I see clients all the time, even when they are not officially in client mode and I am not in work mode. It is just how it is. How could I live here and be blurry? Just seeing me for my whole person is important. I like that y’know.*

### The feeling of always being available as a health worker to community even when not at work

**Fig. 4:**
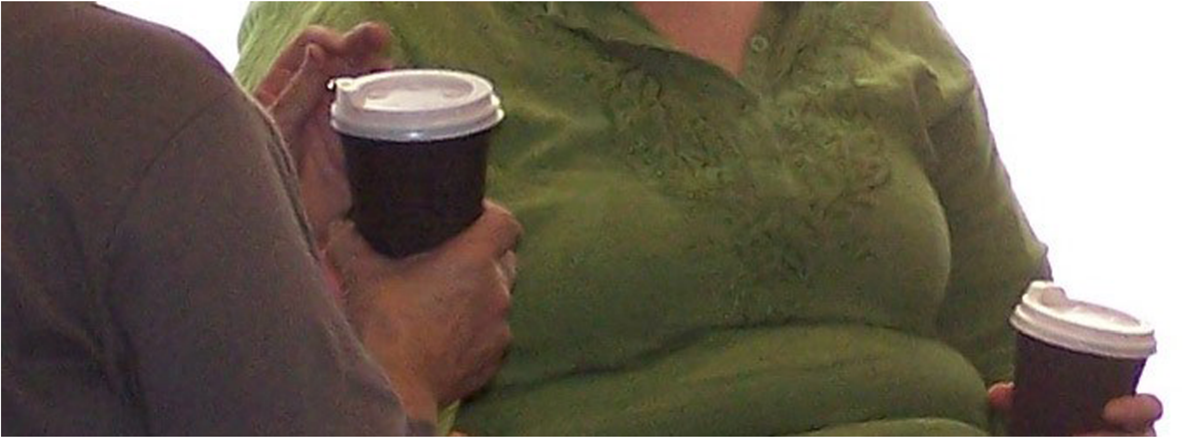
This is not just a coffee

Kim asked a work colleague to take Fig. 4 (*This is not just a coffee*) for her, because she wanted to be in it. She wanted a photo that showed her having a coffee with a client, explaining that she did that all the time, both during work hours and after work hours:

*So this time we [Kim and her client] had gone and bought a good coffee- not just the stuff we normally drink at work. I think it was because this client had reached a big milestone, so the good coffee was a celebration of that. She would have normally celebrated with drugs, so sitting with her and sharing a good coffee was a huge step. Of course she often thinks about celebrating with other things, and I think that is what we were talking about in this photo, actually.*

Kim spoke about how her community saw her health professional identity as part of herself all the time, not just when she was officially at work. Kim did not believe this reflected poor boundary setting, but rather she felt honoured that community saw her that way as she, and other Team Members chose not to hold an impenetrable boundary between the personal and the professional selves:

*Sometimes I am ‘off work’ and a client comes up to me, say in a shopping centre for example, and they are so proud that they have done something good for their health. They want to tell me about it. Imagine if I turned and said ‘Hey how about we talk about that on Monday at the clinic?’…well, they’d think I was not interested, or worse, think I don’t care! And that’s just not me.*

The earlier example (Fig. 1*The royal finger*!) described how another Team Member, Tia, had developed ways of communicating with her client, Kev. Tia welcomed Kev’s approach in the supermarket when she was officially off work; she appreciated that Kev had good news and had already established ways of working with Kev that allowed them both to engage in an out of work context that was safe and appropriate for both of them. Showing a client care and giving them time was important for Kim and Tia; their examples show how they both work with seamlessness by using friendship-like ways of communicating with clients. Both had found a way to provide clients with that time and interest, even when officially “off work”; Tia named exactly how it was for her “I live in a small community, you know, everyone knows everyone” and didn’t see Kev’s approach as a challenge for her. And while Kim was still doing boundary work she was doing it from within a space that was informed by her experience of the Cultural Interface:

*My clients know I live here and they know that they can yarn with me when they see me outside of work. Sometimes we end up having a cuppa like this…talking about their health (which is work stuff for me) but I wouldn’t turn them away. They see me as who I am, and I am their health worker. Sometimes I have had my kids with me, so we don’t stop for a coffee like this, but have a quick yarn up about it. Y’know, respectful listening and a pat on the back – but sometimes a coffee is the way to go.*

### The need to set an example

**Fig. 5:**
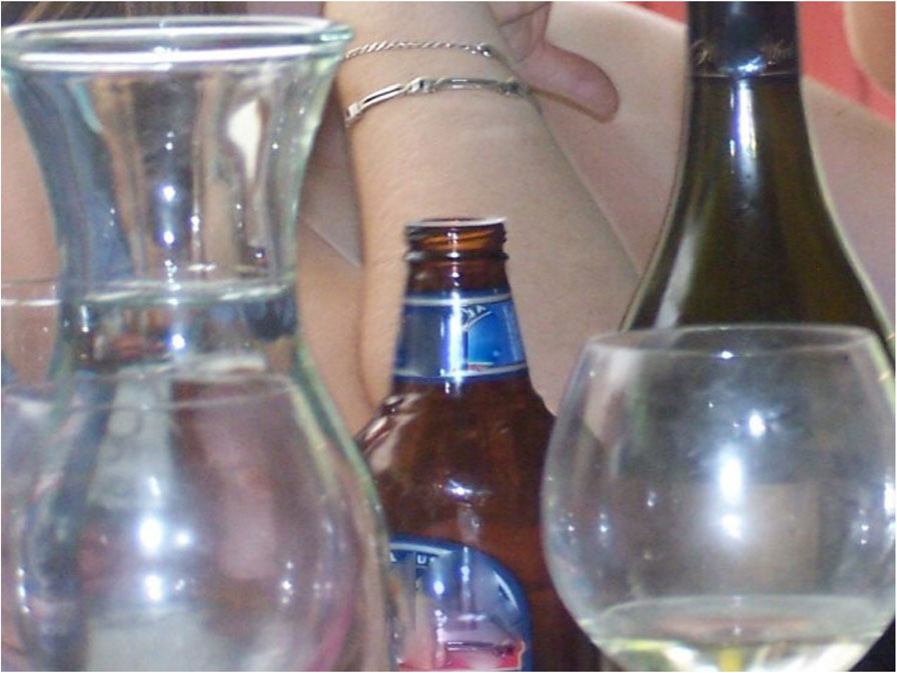
Do what we say, not do

Marlene’s PhotoYarning focused on how she was always aware of her professional identity, even when she was not officially at work. She spoke about how people looked at her as a health professional in community even when she was not at work and how she felt a responsibility to that professional identity. As such Marlene was always aware of what she was doing, even when she socialised. While not always a pleasant way to be, this awareness was a strategy Marlene had developed that allowed her to blend her professional and community identities. Marlene felt that many of her colleagues did not share the same feeling. Marlene used Fig. 5 (*Do what we say, not do*) to PhotoYarn about the ongoing connection between her identity in a professional space and in a social/personal space:

*I took this [photo] at a work do. We were out as a team celebrating something, you know?...But something I thought was real funny. Here we all were- we are all drug and alcohol workers, or nurses or doctors, and the amount of alcohol that was on the table was unreal!... I don’t judge people at all- I just choose not to drink, you know? Clients see me in community and they look at what I am doing… Well, I guess I just thought it was a bit funny. We, our team, work with clients about their drinking habits. We did a lot of work on safe drinking and amounts to drink that keep them safe- and here we were, as a team, doing lots of drinking (lots) and I was wondering I wondered why it was OK for us [to be over drinking] and not OK for our clients?*

Marlene was very concerned about how her work team sees their drinking behaviours as being different to the community’s drinking behaviours, but equally her seamlessness was creating a challenge for her during this work-social event. Marlene was worried that no other colleague appeared to be concerned about clients/community seeing them drinking in large amounts, and that made her wonder about why it worried her so much. Here Marlene adopted a position of role model and held herself to a high standard; she understood the contested space between herself as a health professional who was celebrating with colleagues and herself as a health professional who lived and worked in a community that experienced problems with alcohol. Armed with both sets of knowledge, Marlene developed strategies for being able to accommodate this example of collegial celebration and appropriate role modelling:

*And so I sat there “celebrating in style” and thought about whether we [the team of health professionals] are fake. That worries me. I had to take the photo to remind me of those things I was thinking. Never want to be fake in the work I do- or in anything. As I sat there I got more and more worried as everyone drank more and more.... Well it didn’t sit right with me, it is not how I am seen in community, even if I am not at work! Why didn’t everyone else feel this too?*

Marlene clearly felt that she had a different perspective on seamlessness than her colleagues, however her enactment of seamlessness highlights some challenges for Marlene who, unlike some of her non-Indigenous colleagues keeps “thinking about things at work” even at the end of the working day:

*....I am pretty sure most of the team won’t even think twice about the drinking they did that day. But I think about things like that, because it is who I am….I don’t think I could ever just clock off the job. Y'know what I mean? I mean, I struggle when I hear a work colleague say "That's it I am out of here, not thinking about that until tomorrow". Gee. I just don't think like that. For me, even if it is the end of the day, I seem to keep thinking about things at work… [long pause]… It’s all me, y’know? My work and other bits of my life. They are all me and all connected.*

Within a range of work and social spaces, Marlene’s colleagues were making their own choices about how they individually managed their personal and professional identities. Marlene resolved the contradiction as follows:

*…I waited a while and then I told them I had to go and pick up the kids. Shit, I really didn’t but I just was worried sitting there and needed to leave. Shit, that was being fake, wasn’t it? Pretending I had to pick up the kids. But I couldn’t tell them what I was worried about, ‘cause they’d laugh, or something, and I just didn’t want that. So I left.*

**Fig. 6:**
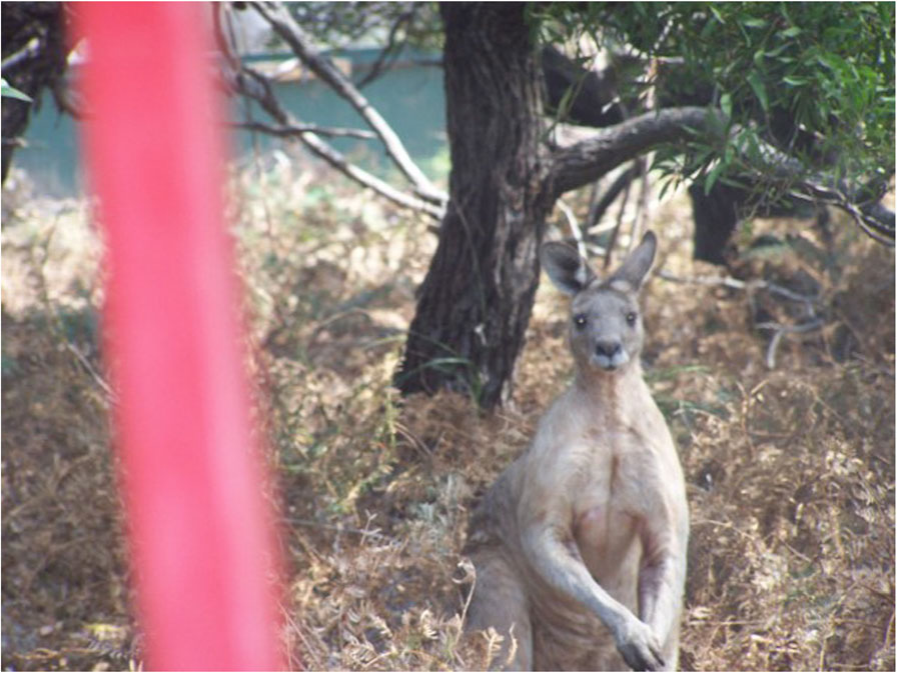
I am watched, always

Pete took Fig. 6 (*I am watched, always*) and PhotoYarned about it, explaining to me that the kangaroo was like the community he lived and worked in:

*Good story here. I took a photo of this old fella- I asked him first! He is important to me, and I think he was sent to make me think on this day I took the photo. I had just had a work mate tell me that he didn’t understand how I managed my personal stuff outside work. He wanted me to go for drinks at the local and I said no because I know lots of our clients drink there. He said I shouldn’t put my clients first. Well, I do put my clients first, it’s how it is for me. Anyway, I went off for a walk at lunch and saw this big ‘roo. And I thought to my self- That’s it. My community are always watching me, just like this big roo was watching me on my walk.*

Pete was acutely aware that he remained visible to community members (who were also health service clients) even when not at work; Pete lived and worked at the Cultural Interface. His knowledge of that space informed his decision not to participate in certain social activities in “out of work” hours. Pete struggled because his work colleague did not understand the decisions he was making from living and working in the Cultural Interface; Pete acknowledged “community always see me and watch me” and had developed a level of comfort about that, stating “it’s not a big deal for me”:

*And I know they see me all the time, they know I work in the health clinic and so I feel that need to be responsible with my own life and health. Imagine if I went off boozin’ up and my clients saw me that way. Well, I would feel like I was letting them down, like I was a fake because I was telling them to be as healthy as they could be, and then they see me drinking on and on… just like this ‘roo, community always see me and watch me. I know that. It’s not a big deal for me, but my work mate just didn’t get it. He thought I was being rude not going for drinks.*

Pete preferences his own way of working over the choices his work colleagues make. Pete’s strategy for working with seamlessness it is be mindful of “always being watched’ and as such he makes choices that he believes enables and supports his ways of working and living in the same community. Pete does not see the position or perspective taken by his colleague who chooses to maintain a personal self (who enjoys going out for drinks) and a professional self who encourages people not to drink (dangerously). Like Marlene, Pete referred to working seamlessly as not being “fake”, being real and not hypocritical. This suggests Pete and Marlene practice consistency and authenticity in their work through working with seamlessness.

## Discussion

The aim of this study was to explore how Australian Aboriginal and Torres Strait Islander health professionals juggle the challenges of working and living in the same community in ways that are positive for both themselves and their clients. While Team Members faced complex identity and role-based challenges, they developed strategies that empowered them to live and work in the same space. As a qualitative study this research undertook an in-depth exploration of the lived experiences of a small group of Aboriginal and Torres Strait Islander health professionals. The study design did not seek a large sample size, but rather sought richness of lived experience data. It did not seek to offer generalisability but rather to offer insight into some strategies used by the Team Members as they lived and worked in their Aboriginal and Torres Strait Islander communities.

Team Members established a comfortable, seamless connect between their professional identities and personal/community identities which I have called the ‘seamless working self’. They described being able to work in a seamless way as meaning they were able to maintain their responsibilities and roles as both Aboriginal and Torres Strait Islander community members and Aboriginal and Torres Strait Islander health professionals. Team Members provided examples of what this seamless connect involved, citing four main aspects: being fellow members of their cultural community, the feeling of always being visible to community as a health worker, the feeling of always being available as a health worker to community even when not at work, and the need to set an example. They described having worked hard to overcome the many challenges they faced as they work and live in their own communities and detailed the strategies they developed to allow them to work this way.

The research I citied in the background section of this article provided a foundation for these findings, especially regarding some of the identity complexities faced by Aboriginal and Torres Strait Islander Team Members who were living and working in the same community. Nakata’s Cultural Interface Theory [34, 36] helped me understand how Team Members navigated the space between working with a patient (in a work context) and maintaining a connection with the same person, in a community context. The data in this study highlight that navigating identity often depends on the context and relationships attached to each role. As such, Team Members adopted an Indigenous Standpoint [39, 63, 64] as they worked and lived in their communities and it is from this theoretical positioning that the data in this article is best understood. Team Members did not position themselves by ‘role’ nor did they attempt to privilege one ‘role’ over another in order to decide how best to work and to live. As they worked in the Cultural Interface they used their Indigenous Standpoint as a tool for empowerment; they knew both the professional space and the community/cultural space they lived and worked within and were able to develop strategies that enabled them to work seamlessly. They operated in the Cultural Interface and used two-way knowledge to develop ways of being, knowing and doing that were comfortable in both their work and living spaces.

This study highlights that, while Team Members identified strongly with their professional role, they did not rank that role as more important than their community role and as such allowed their professional identity to be influenced by their community identity, and vice versa. This positioning is well supported by Indigenous Standpoint Theory [39, 40, 63], with Team Member’s enacting their Aboriginal and Torres Strait Islander identities (and cultural protocols and processes) as they worked at the Cultural Interface. Team Members developed a “seamless working self” that engaged Aboriginal and Torres Strait Islander ways of being, knowing and doing while challenging some of the dominant practices and processes in their workplaces. The strategies developed by Team Members reflect working in the Cultural Interface; while that space is understood by the Team Members, it is a space that could be better understood and valued by the dominant health system and by non-Indigenous colleagues who hold different perspectives on how to effectively live and work in the same community.

Lander’s definition of identity as “contextually specific, fluid, a conjoint construction created … in interaction with others” ([33] p34) more accurately describes how Team Members in my study negotiated their professional and personal/community identities with seamlessness and aligns with Indigenous Standpoint Theory [36, 38–41]. This helps us understand how Team Members enact their Aboriginal and Torres Strait Islander ways of being, knowing and doing in the work they do with clients as they achieve no separation between the work they are doing as a health professional as they work in community while living in the same community.

This study shows that Team Members’ seamlessness also involved working with friendship-like ways [49] of working to uphold personal boundary work with clients and that difference was best achieved through developing seamless working ways. Team Members’ reported a feeling of always being watched, of being aware of working in the Cultural Interface between dominant work protocols and practices (for example, leaving work at work at the end of the day) and community protocols and practices (using empathy and actively engaging with other community members regardless of the context, being available for community). Their awareness of this space, their seamlessness, afforded Team Members the privilege of making choices and developing strategies so they could uphold both spaces, ultimately having developed a level of comfort about working with seamlessness, as highlighted by one Team Member who said “it’s not a big deal for me”. Being able to uphold both spaces (living and working in the same community) was important to Team Members because they were aware of, and valued, both cultural *and* professional obligations, roles and responsibilities. Team Members valued being a fellow member of the cultural community in which they also worked and expressed not wanting to “let them [cultural community] down”. Such a deep engagement with cultural, social and political responsibility seems to obligate Team Members to develop ways of working that were seamless, as noted by Team Member, Marlene: “My work and other bits of my life. They are all me and all connected”. This obligation was not seen as a negative factor, but just as a given “I do put my clients first, it’s how it is for me” (Pete, Team Member).

My findings also highlighted how Team Members’ roles and responsibilities, in addition to their clinical and health-based roles, meant that they maintained close connections with community cultural, social and political knowledge [13, 10]. Team Members spoke of always being aware of their professional identity, even when they were not officially at work. They reported that people always looked at them as a health professional in community and how they felt a responsibility to that professional identity. Being aware of this was a strategy Team Members had developed that allowed them to blend their professional and community identities in a positive, role-modelling capacity rather than seeing that position as a burden.

This study presents findings that suggest Team Members have found ways to allow their professional and personal/community identities to coexist, allowing them to work and live in the same community with seamlessness; these strategies included using empathy as they communicated with clients, establishing and valuing boundaries that were understood by clients and the Team Members themselves and developing friendship-like connections with clients [49, 61]. Literature presented earlier in this article identified both benefits and challenges in maintaining multiple workplace connections and relationships [42, 43]. While my findings align with this literature, they differ in specifically reporting the benefits and challenges of maintaining multiple relationships and connections when working in a context where Team Members (health professionals) were also members of other social and cultural parts of the same Aboriginal and Torres Strait Islander community.

This study describes how a group of Aboriginal and Torres Strait Islander health professionals worked hard to overcome the many challenges they faced as they work and live in their own communities and detailed the strategies they developed to allow them to work this way without burning out. While some literature describes a risk of burnout when Aboriginal and Torres Strait Islander health professionals do not have ‘down time’ from living and working in the same community [65–67], at the time of conducting this research, Team Members identified strategies to allow them to work with a comfortable, seamless connect between their work and community roles and not burn out. While creating this seamlessness was not easy, Team Members considered it an important feature of the work they did and vital if they were to be able to provide quality health service to their community.

Team Members did not provide examples of feeling burnt out; instead they spoke about the strategies they had developed and established that facilitated their work with clients while also keeping them functional and healthy members of a workforce and a community. However, they reported that the seamless working self was at odds with the way many of their non-Indigenous Australian colleagues worked and it was not well understood. Team Members were not being considered unprofessional because of their seamless working ways; rather they felt that their colleagues and managers saw them as always being too close to work and clients, implying that was not good for them at a personal level. However, the lens through which Team Members viewed their seamless ways of working is different to the viewing lens of (most) of their non-Indigenous colleagues. Team Members cultural lens highlighted their high levels of cultural and community responsibility and called on community values of reciprocity, giving and sharing; those values greatly influenced Team Members’ working with seamless ways.

## Conclusion

While it cannot be assumed that establishing and maintaining boundaries is clean and simple if the health professional lives and works in different communities, the findings in this study suggest that, when living and working in the same community, cultural connection is one important contributing factor to being able to develop ways of working with seamlessness as it provided Team Members with a strength and a status that supported their work; one Team Member reminding me that working with seamlessness was about “the cultural connection to her community [that] committed her to working that way”.

A major concern expressed by Team Members in this study was the lack of understanding their colleagues and workplaces had about the way Team Members worked, largely reflecting a lack of ability to see or appreciate more than one world view. If Team Members’ managers and colleagues were encouraged to develop an understanding of how Team Members work at the Cultural Interface Team Members could potentially reap the emancipatory effects as their colleagues expand their world view and interpretations to include other people’s world views and ways of being, doing and knowing [38]. As such, I argue for health services to better understand and value the friendship-like [61] connections Team Members develop and engage in their work with clients. Team Members in this study adopted practices with clients that they felt comfortable with; they developed ways of being at home in community that they felt comfortable with. At the same time, they sought to perform their professional role with care, professionalism and cultural safety while keeping client service provision and care at the focal point.

We need to attend to this disconnect between Aboriginal and Torres Strait Islander health professionals’ experiences and perspectives and their workplaces. The rhetoric of trying to addressing this disconnect is evident in the National Aboriginal and Torres Strait Islander Health Plan [68] as it proposes how health systems can better value and come to understand Aboriginal and Torres Strait Islander ways of working in health to enhance health service provision for Aboriginal and Torres Strait Islander clients and Aboriginal and Torres Strait Islander health staff. The plan speaks of making change to models of care, to workplace management and supervision, of building capacity and capability of the health workforce – of making institutional and systemic change. However, my findings reflect Team Members’ experiences of not having their Aboriginal and Torres Strait Islander ways of working valued or understood. Their ways of working were very evidently not seen to “optimize their contribution as individuals to the health workforce and to strategies to achieve Aboriginal and Torres Strait Islander wellbeing” ([68] p23). If we are committed to supporting, valuing and embracing the work being done by Aboriginal and Torres Strait Islander members of the health workforce, then we need further research to understand the factors contributing to collegial and managerial opposition to the reality of Aboriginal and Torres Strait Islander health professionals’ ways of work.

## Data Availability

The datasets generated and/or analysed during the current study are not publicly available to ensure the upholding of the study’s granted ethical approval. Given the personal and cultural nature of some of the data, the ethical approval was provided for data to be used and analysed for this study and not to be provided publicly.

## Abbreviation

HREC: Human Research Ethics Committee

## Footnotes

1 Australian Aboriginal and Torres Strait Islander peoples are the original inhabitants and custodians of the land now known as Australia.

2 As an Aboriginal woman from New South Wales I call myself Koori.

3 Throughout this study ‘research participants’ were named Team Members. This term represents the true engagement between me (‘researcher’) and Team Members (‘research participants’) throughout the whole research process.

4 Indigenous refers to First Nations peoples of the world.

5 Tia is a pseudonym, chosen by this Team Member. Each Team Member chose a pseudonym, and these are used throughout the manuscript.
